# Attitudes to *in vitro* meat: A survey of potential consumers in the United States

**DOI:** 10.1371/journal.pone.0171904

**Published:** 2017-02-16

**Authors:** Matti Wilks, Clive J. C. Phillips

**Affiliations:** 1 Early Cognitive Development Centre, School of Psychology, University of Queensland, Brisbane, Australia; 2 Centre for Animal Welfare and Ethics, School of Veterinary Science, University of Queensland, Gatton, Australia; U.S. Geological Survey, UNITED STATES

## Abstract

Positivity towards meat consumption remains strong, despite evidence of negative environmental and ethical outcomes. Although awareness of these repercussions is rising, there is still public resistance to removing meat from our diets. One potential method to alleviate these effects is to produce *in vitro* meat: meat grown in a laboratory that does not carry the same environmental or ethical concerns. However, there is limited research examining public attitudes towards *in vitro* meat, thus we know little about the capacity for it be accepted by consumers. This study aimed to examine perceptions of *in vitro* meat and identify potential barriers that might prevent engagement. Through conducting an online survey with US participants, we identified that although most respondents were willing to try *in vitro* meat, only one third were definitely or probably willing to eat *in vitro* meat regularly or as a replacement for farmed meat. Men were more receptive to it than women, as were politically liberal respondents compared with conservative ones. Vegetarians and vegans were more likely to perceive benefits compared to farmed meat, but they were less likely to want to try it than meat eaters. The main concerns were an anticipated high price, limited taste and appeal and a concern that the product was unnatural. It is concluded that people in the USA are likely to try *in vitro* meat, but few believed that it would replace farmed meat in their diet.

## Introduction

Recent times have seen a growing awareness surrounding the negative repercussions associated with the production and consumption of meat products [1]. These focus primarily around environmental outcomes, such as greenhouse gas emissions [2]; ethical concerns for the animals raised under intensive farming conditions [3]; and the inefficiency of meat production in terms of resource use and the capacity to feed an ever increasing world population [4]. Despite the growing recognition of these concerns in the USA, meat consumption there is at three times the global average [5] and also rose 5% in 2015 –a jump larger than any that had been seen since the 1970s [1]. Moreover, we often see that meat consumption is equated with masculinity; indeed vegetarian men may be perceived as less masculine than omnivorous men, with vegans being seen consistently as less masculine [6, 7].

In the light of people’s continued desire to eat meat, it seems the problems associated with consumption are unlikely to be fully resolved by attitude change. Instead, they must be addressed from an alternate perspective: changing the product. Researchers in the Netherlands have examined possible options to do this; developing a product from cultured animal-derived stem cells in the laboratory (*in vitro* meat, IVM)[8, 9]. In April of 2013 they cooked and ate the world’s first laboratory grown meat patty, an *in vitro* hamburger [10], and are currently aiming to develop this product as a viable non-farmed alternative for future meat production [8, 9]. In doing so, they aim to alleviate some of the aforementioned ethical and environmental concerns surrounding farming practices [11], and address the increasing global demand for meat [12].

According to the leading scientist involved in the production of IVM, ‘detailed studies are needed to gain more insight into potential psychological obstacles that could lead to rejection’ [8]. Given the American public’s general reluctance to sacrifice meat from their diet, this new approach to meat production may be met with some apprehension. Since the development of the IVM concept, research has examined the practical and philosophical components of IVM [13–15]. However, thus far, public perception remains relatively unexamined.

Several qualitative analyses have been conducted examining people’s perceptions of IVM in comments from online news sources [16], as well as in a group forum and interview context [17–19]. In general, positive perceptions have been related to public health and the potential environmental benefits, while negative themes focused around the unnatural and unappealing qualities of the product, safety and feasibility of industrial production. Generally, people seemed willing to try the product, but were reluctant to engage further [18]. Moreover, one study found that geographical location was related to positivity—whereby those who lived in rural locations held more negative views about the product. However, people who make comments online tend to hold more extreme views [20], indicating that the comments identified in the online comment analyses may illustrate stronger opinions than would be represented in the general public.

In terms of quantitative analyses, two online surveys have been conducted. One study [21] surveyed participants in Belgium, using a convenience sampling method which produced participants that were younger and more highly educated than the general public. This may have resulted in left-orientated sample bias [22]. The second survey, conducted by researchers in the Netherlands in 2013 surveyed a large number of participants, with no convenience sampling reported [8]. Both surveys found that the majority of participants were not familiar with the concept; however, around three quarters of each sample said that they would try IVM. The positive factors associated with IVM were the reduction of waste and suffering [21] and resolving the world food problem [8, 9, 21]. When considering barriers to IVM consumption that the primary concern for those reluctant to try IVM was the genetically engineered nature of the product, aligning with previous research on IVM which has identified concerns around its naturalness [16, 19, 21]. Price was also identified as a barrier across both studies, with the majority of participants being unwilling to pay more than for traditionally produced meat [8, 21].

Overall, attitudes towards IVM appear mixed, and would also be influenced by practical factors, such as price, and perceptual factors, such as naturalness. However, the current literature is not yet able to convey the full story. The majority of research has used qualitative measures, which, while informative, limit the capacity to understand large scale perceptions. Of the quantitative research, some studies are affected by sampling bias, and moreover, limited research has attempted to understand the mechanisms for resistance. To further our understanding of the public perception of this product, attitudes to comparable products must be examined. Genetically modified (GM) food production research is of relevance. The use of GM foods is longstanding and extensive, with many products now modified [23]. However, the general opinion towards GM across the world remains negative [24, 25]. Moreover, public opposition persists despite people’s conscious lack of knowledge about the product [26]. A meta-analysis has identified a number of factors that consistently associate with opposition, including higher perceived risks than benefits, little trust in institutions and moral concerns [27].

In contrast, initial perceptions of IVM have been positive—though several barriers have been identified in regards to concerns about naturalness, genetic engineering and the potential cost [8, 21]. The disparities between perceptions of the two products are potentially a product of methodological differences between studies. However, they may also be attributable to the conceptual differences between IVM and GM food production; relating to potential to alleviate environmental and animal welfare concerns. Across several studies, animal welfare is consistently identified in the top three primary motivators for choosing to abstain from meat, with health and environmental concerns also being cited [11]. If publically accessible, IVM has the capacity to provide a meat source that does not rely on farming, and thus does not raise the same ethical and environmental concerns associated with traditional farming [8, 9]. This unique component is essential to explore if we are to understand both the support and potential barriers to IVM being an accepted meat production method of the future.

The potential for cross cultural variation in perception must also be explored. Research demonstrates that GM food perceptions vary by country, with those in the United States of America (USA) being more positively disposed to GM food than Europeans [24, 25] The majority of research examining perceptions of IVM has been in Europe [8, 18, 21], with one study conducted in New Zealand [17]—raising the question as to whether non-European participants have differing perceptions. Given the disparity of results, methodological issues, and the limited cross cultural sampling in the literature to date, research is needed to objectively explore attitudes towards IVM as a potential meat production alternative for the future. To address this, the current study aims to investigate perceptions of IVM in the USA, to better understand the potential for this product to be engaged by the public in a country generally supportive of innovation and a major potential market.

## Method

We studied perceptions of cultured meat through an online survey administered between March and June of 2016. Given that past research on this topic has been subject to sampling bias, we sought to collect responses from a representative sample. Amazon Mechanical Turk (mTurk) is an online crowd surfing marketplace that connects researchers with participants. Researchers advertised their experiment or survey online and participants, who have previously signed up to participate in research via this platform, have the option to select whichever experiment or survey they would like to participate in. Participation is entirely anonymous and voluntary, and participants can withdraw at any time without prejudice. Participants are reimbursed for their participation (in this case, US $0.50). This platform has been shown, by a number of studies, to collect data that is at least as reliable as those obtained via traditional methods, as well as providing a significantly more diverse sample than typical American college samples or other online recruitment [28–31]. In using mTurk as our data collection process we were able to include eligibility criteria of respondents being 18 years or older, and living in the United States. We chose the United States as the site for this survey because in this country there are concerns about the ethics of eating meat, a high level of innovation to develop products such as IVM and also a ready potential to collect data through MTurk.

First, participants were asked their age, gender, education level, political affiliation (2 questions), income per annum and two questions about their meat consumption (categorical rating and percentage of diet that is made up of meat) (S1 Questions). Following this, participants were asked about their familiarity with IVM. Regardless of their rated knowledge, all participants were provided with a short informative text about the product (S1 Questions), which was designed to be objective and to inform, rather than influence, participants before participation.

Participants were asked a number of questions about how they perceived IVM relative to meat produced by farming (referred to here and elsewhere as ‘farmed meat’). These questions were presented on a Likert-type scale ranging from 1 (much more) to 5 (much less). Following this, participants were asked to imagine that IVM was commercially available and whether they would be willing to try it, again on a Likert-type scale, 1 (definitely yes)—5 (definitely no). If participants selected options from 1–4, they were asked a number of follow-up questions examining their willingness to purchase, try and consume this product, as well as how much money they would be willing to pay relative to farmed meat. All questions were presented on a 1–5 Likert-type scale. If participants indicated that they would not try IVM, these follow up questions were not presented. All participants were then given a multi-response option investigating under what conditions they would be unwilling to try IVM. This question also had an open ended response option. Participants were then given two more multi-response questions asking which types of meat they currently eat, and which types of meat they would be willing to eat if it was produced via *in vitro* methods. Finally, participants were asked to rate how much they agreed with a number of statements, on Likert scales of 1 (strongly agree)—5 (strongly disagree), which gauged their opinions on statements that had been identified in past research as positive or negative potential outcomes of IVM production.

### Statistical analysis

Data was analysed using Minitab Version 16. Analyses were primarily logistic regression, both with binary and multivariate outcome variables. Input variables were age, political affiliation (2 questions: left to right, and liberal to conservative) and percentage of meat in diet as continuous variables, and gender, income, education level and eating habits as categorical variables. The logistic regression models used a logit link function. Differences in the proportion of participants reporting that they ate farmed meats and that they would eat IVM of each of the major type of meats was analysed in pairs of meats by Fisher’s Exact test, a modification of the Chi Square test appropriate if there are cells with values less than 5 counts.

## Results and discussion

This study was granted ethics approval within the School of Psychology Ethics Review Process at the University of Queensland, approval number 16-PSYCH-PHD-20-AH. This work has provided the first large scale examination of attitudes in the USA towards IVM, identifying initial perceptions as well as barriers to engagement. A total of 673 participants were included in the survey (328 male, 340 female, 5 other), with an age range of 18 to 70 (*M* = 32.58, *SD* = 10.79). Four extra participants did not complete the survey, with one opting out before beginning and three not meeting the eligibility criteria (under 18). Compared to the national US data [32], our participants were slightly younger (M = 5 years), had a slightly lower income and were and more likely to have an undergraduate degree (Table 1). However, the data is still broadly representative of the population, and thus should provide insight into general US attitudes (S1 Dataset).

**Table 1:** Demographic responses of the current sample, compared to the national data, where available [32].

**Categorical Variables**			
**Question/response options**	**No. of Responses**	**% of sample**	**% US National Data**
			
**Gender**			
**Male**	328	48.0	49
**Female**	340	49.7	51
**Other**	5	.07	
**Income**			*
**Less than $20,000**	200	29.2	10.6
**$21,000 - $39,000**	166	24.7	17.1
**$40,000 - $59, 000**	135	20.1	15.1
**$60,000 - $79,000**	82	12.2	13.2
**$80,000 - $99,000**	42	6.2	10.6
**More than $100,000**	48	7.1	33.4
**Education**			
**No education**	1	0.1	0.3
**Primary school**	0	0	3.9
**Some high school**	11	1.6	3.3
**Completed high school**	198	28.9	29.6
**Technical qualification or trade certificate**	63	9.2	4.1
**College/undergraduate degree**	305	44.6	24.4
**Postgraduate degree**	93	13.6	10.6
**Eating habits**			
**Meat eating**	602	88	
**White meat only**	21	3.1	
**Pescatarian**	7	1.0	
**Vegetarian**	19	2.8	
**Vegan**	14	2.0	
**Other**	9	1.3	
**Continuous Variables**			
**Question**	**Mean**	**SD**	**Mean US National Data**
**Age**	32.5	10.8	37.3
**Political affiliation**			
**Left—right**	4.3	2.1	
**Liberal—conservative**	4.1	2.2	
**% diet consisting of meat**	39	19.5	

*US data was only available for family income, as opposed to individual income.

### Willingness to engage

Results suggest that people are somewhat willing to engage with IVM (Table 2). Nearly two thirds of the sample would try IVM (probably or definitely), with only around one fifth stating they would not try it (probably or definitely). However, willingness to eat regularly was reduced, with approximately one third of the sample being willing to eat IVM regularly or as a replacement for farmed meat (probably or definitely). This suggests that, while the majority of the sample are willing to try, there are stronger reservations around fuller engagement.

**Table 2:** Participants’ willingness to engage with the product.

Question/response options	No. of Responses	Percentage of Sample
**Would you be willing to try IVM?**		
**Definitely yes**	213	31.1
**Probably yes**	234	34.2
**Unsure**	80	11.7
**Probably no**	86	12.6
**Definitely no**	58	8.5
Would you be willing to eat IVM regularly?*		
**Definitely yes**	44	6.4
**Probably yes**	179	26.2
**Unsure**	211	30.8
**Probably no**	129	18.9
**Definitely no**	51	7.5
Would you be willing to eat IVM as a replacement for farmed meat?*		
**Definitely yes**	49	7.2
**Probably yes**	166	24.3
**Unsure**	180	26.3
**Probably no**	144	21.1
**Definitely no**	62	9.1
**Not applicable (I do not eat farmed meat)**	13	1.9
How willing would you be to eat IVM compared to soy substitutes?*		
**Much more**	132	19.3
**Somewhat more**	194	28.4
**Neither more nor less**	151	22.1
**Somewhat less**	101	14.8
**Much less**	36	5.3
How much would you be willing to pay for IVM compared to farmed meat?*		
**Much more**	7	1.0
**Somewhat more**	101	14.8
**Neither more nor less**	230	33.6
**Somewhat less**	198	28.9
**Much less**	78	11.4

Participants who reported ‘definitely not’ willing to try IVM were excluded from the subsequent questions (*).

### Potential barriers to engagement

A number of potential barriers to engagement were identified, and coded into 9 categories.: The proportion of respondents citing these reasons were: taste/appeal of the product 79% (n = 535), ethical concern 24% (n = 168), price 20% (n = 137), health concerns 4% (n = 31), safety concerns 3% (n = 23), religious reasons 3% (n = 22), environmental concern 1% (n = 7), and economic impact <1% (n = 4). Therefore, it appears that taste/appeal is the primary barrier, followed by ethical concern and price. In regards to price, participants were reluctant to pay more for IVM compared to farmed meat, in general. Approximately one third of the sample was willing to pay neither more nor less for IVM relative to farmed meat (34%), similar to those who would pay somewhat (29%) or much (11%) less. A smaller proportion of the sample was willing to pay somewhat more (15%), while few participants (1%) were willing to pay much more. These findings align with previous research suggesting that taste/appeal is a key barrier to engaging in alternative meat consumption practices [17].

### Types of meat

We examined the proportion of participants responding that they eat each of the main type of meats and compared this with the proportion of participants that reported they would eat each type of IVM (Table 3). Nearly all respondents (80–90%) indicated that they currently ate fish, poultry, pork/bacon/ham and beef, but not horse or dog/cat. Respondents said they were most unlikely to eat fish if produced as IVM, but were also less likely to eat poultry, pork/bacon/ham and beef if produced as IVM. By contrast they said that they were more likely to eat horse or dog/cat if produced as IVM than as farmed meat. This aligns with Western conceptualisation of food and non-food animals [33]. Interestingly, the reversed trend here (where people became more willing to eat non-food meats such as horse, dog and cat) suggests that the stigmatization might be related to the animal being alive [34]. It should be noted, though, that due to a small number of positive responses in the non-food meat columns, further research would be required to examine this effect. As nearly half the sample stated that they would be likely to eat IVM compared to soy substitutes, attitudes to IVM may be considered positive relative to soy products.

**Table 3:** Types of meat currently eaten, and willing to be eaten if produced via *in vitro* methods. Means with different superscripts are significantly different by Fisher’s Exact Test.

Type of meat	Farmed meat	IVM	Difference
**Fish**	548 (80.3)	308 (45.0)	-35.24^a^
**Poultry**	611 (89.5)	430 (62.9)	-26.61^b^
**Pork/Bacon/Ham**	558 (81.7)	410 (69.1)	-12.64^b^
**Beef**	592 (86.5)	482 (70.6)	-15.93^b^
**Horse**	0 (0.0)	36 (5.3)	+5.27^c^
**Dog/Cat**	1 (0.1)	21 (3.1)	+2.93^c^

### Perceptions of IVM

#### Compared to farmed meat

We examined general perceptions of IVM, both in comparison to farmed meat (Table 4), as well as general perceptions based on themes identified in past research (Table 5). Respondents felt that IVM was less natural, less appealing, less tasty than farmed meat, but more environmentally friendly and with less risk of zoonosis compared to farmed meat (Table 4).

**Table 4:** Mean perceptions of IVM compared to farmed meat (1 much more—5 much less).

Question	Mean	SD
**How healthy do you think IVM is compared to farmed meat?**	3.08	0.95
**How natural do you think IVM is compared to farmed meat?**	4.29	0.83
**How environmentally friendly do you think IVM is compared to farmed meat?**	1.97	0.97
**How ethical do you think IVM is compared to farmed meat?**	2.20	1.14
**How appealing do you think IVM is compared to farmed meat?**	3.71	1.19
**How tasty do you think IVM would be compared to farmed meat?**	3.76	0.84
**How much of a risk do you think there is for zoonosis for IVM compared to farmed meat?**	3.95	1.02
**On a global level, to what extent do you think meeting demand for meat using IVM would be cheaper or more expensive than farmed meat?**^1^	3.17	1.32

^1^ Much less expensive than farmed meat—5 much more expensive than farmed meat.

**Table 5:** Agreement with statements about attitudes towards IVM, as identified from past research (1 strongly agree—5 strongly disagree).

Question	Mean	SD
**IVM is unnatural**	2.08	1.02
**IVM is disrespectful to nature**	3.69	1.19
**IVM will reduce the number of happy animals on earth**	4.07	0.93
**IVM will encourage the possibility of cannibalism**	4.17	1.08
**IVM is ethical**	2.41	1.09
**IVM will improve animal welfare conditions**	2.29	1.03
**IVM will be able to solve world famine problems**	2.53	1.03
**In the future, IVM will be a viable alternative to farmed meat**	2.42	1.01
**IVM will reduce the impact of global warming associated with farming**	2.55	1.18
**The production of IVM will have negative impacts on traditional farmers**	2.03	0.94

#### General perceptions

Respondents generally agreed that IVM is unnatural, again demonstrating concerns around naturalness, as has been found in previous literature [16, 19, 21]. However, participants also agreed with a number of positive factors, including that IVM would improve animal welfare conditions. Moreover, there was some agreement that IVM was ethical, a viable alternative to farmed meat, has the potential to solve world famine problems and reduce the impact of global warming associated with farming. Respondents disagreed that IVM was disrespectful to nature or that it would reduce the number of happy animals on earth. The logic behind the latter concept is that production of IVM might replace intensively farmed animals, about which there is greatest concern by the public, leaving livestock in extensive farming systems that arguably have better welfare. However, respondents, on average, also felt that it would have negative effects on traditional farmers. The concept that IVM might increase the possibility of cannibalism gained little credence. Therefore, we can see that while there are some general concerns with IVM, both independently and relative to farmed meat, participants were able to identify positive outcomes associated. However, as identified in previous research, [19], these positive outcomes tend to be distal, while the concerns are more proximal.

### Predictive factors: Demographics

Three demographic factors were found to be generally predictive: gender, political affiliation and current meat consumption. Highest level of completed education was not found to be predictive of responses, and we excluded it from the model for 7 questions as inclusion prevented the model from converging (S2 Questions).

#### Gender

Gender was found to be the most significant demographic predictor, showing differences on 66% of questions (16/24) (Table 6). Generally, compared with women, men were more receptive to IVM. Men were found to be more willing to engage with IVM as a product, and had more positive views of the product, with the exception of two questions. This might be reflective of the aforementioned current attitudes towards meat consumption, such that eating meat is identified as a masculine practice [6, 7]. This would indicate that the perceptions of IVM are not unique compared to farmed meat, in this context. However, it should be noted that while gender was consistently a significant predictor of response, the effect sizes identified were small—with gender predicting only small differences. As such, interpretation should be cautious.

**Table 6:** Mean male and female responses to the IVM survey that were significantly (P < 0.05) affected by gender, with regression coefficients, P values, Odds ratios and confidence intervals. Questions range from 1–5 (1: much more/strongly agree/definitely yes—5: much less/strongly disagree/definitely no).

Question	Male	Female	Coefficient (β)	P Value	Odds Ratio	95% CI
**Have you heard of IVM? (1 yes, 2 no)**	1.78	1.82	.742	< .01	2.099	1.46–3.02
**How healthy do you think IVM is compared to farmed meat?**	2.99	3.16	-.403	.007	0.67	0.50–0.90
**How environmentally friendly do you think IVM is compared to farmed meat?**	1.83	2.10	-.73	< .001	0.48	0.36–0.65
**How ethical do you think IVM is compared to farmed meat?**	2.13	2.27	-.36	.012	0.69	0.52–0.92
**How appealing do you think IVM is compared to farmed meat?**	3.55	3.87	-.081	< .001	0.44	0.33–0.60
**How tasty do you think IVM is compared to farmed meat?**	3.66	3.86	-.66	< .001	0.51	0.38–0.69
**Would you be willing to try IVM**	1.97	2.63	-1.15	< .001	0.32	0.23–0.43
**Would you be willing to eat IVM regularly?**	2.71	3.18	-.89	< .001	0.41	0.30–0.56
**Would you be willing to eat IVM as a replacement for farmed meat?**	2.96	3.19	-.52	< .001	0.59	0.44–0.80
**How much would you be willing to eat IVM compared to meat substitutes?**	2.25	2.83	-.95	< .001	0.38	0.28–0.52
**IVM is unnatural**	2.05	2.12	.53	< .001	1.71	1.27–2.30
**IVM is ethical**	2.44	2.39	-.53	< .001	0.58	0.44–0.78
**In the future, IVM will be a viable alternative to farmed meat**	2.37	2.45	-.56	< .001	0.57	0.42–0.76
**IVM will reduce the impact of global warming associated with farming**	2.58	2.51	-.34	.018	0.71	0.53–0.94
**The production of IVM will have a negative impact on traditional farmers**	2.05	2.03	.52	< .001	1.68	1.25–2.25
**IVM is disrespectful to nature**	3.59	3.78	.55	< .001	1.75	1.31–2.33
**IVM will encourage the possibility that humans could be eaten i.e. cannibalism could occur**	4.21	4.14	-.367	.017	0.69	0.51–0.94

#### Political affiliation

Political affiliation, which was assessed as liberal/left and conservative/right, was consistently predictive of responses. People who identified as liberal were likely to see IVM as more ethical [β = -.15, *p* = .03, OR = 0.86, (95% CI, 0.75 to 0.98)], natural [β = -.195, *p* = .008, OR = 0.82, (95% CI, 0.71 to 0.95)], and tasty [β = -.14, *p* = .044, OR = 0.87 (95% CI, 0.76 to 1.00)] than those who identified as politically conservative. Liberal identifiers were also more willing to eat IVM regularly [β = -.15, *p* = .040, OR = 0.86, (95% CI, 0.74 to 0.99)], and as a replacement for farmed meat [β = -.16, *p* = .028, OR = 0.85, (95% CI, 0.73 to 0.98)] than conservative identifiers, and were willing to pay somewhat or much more for IVM than regular meat compared to conservative responders [β = -.25, *p* = .001, OR = 0.78, (95% CI, 0.67 to 0.90)]. Liberal identifiers were more likely to agree that IVM could reduce the impact of global warming associated with traditional farming [β = -.158, *p* = .020, OR = 0.85, (95% CI, 0.75 to 0.98)], while conservative identifiers were more likely to agree that IVM would have a negative impact on traditional farming than liberal identifiers [β = .16, *p* = .021, OR = 1.18, (95% CI, 1.02 to 1.35)].

Being politically left or right also predicted a number of responses. As with being liberal or conservative, those who identify on the political left were more willing to try IVM than those who identify on the political right [β = -.15, *p* = .032, OR = 0.86, (95% CI, 0.75 to 0.99)]. Those on the political left were more likely to agree with the statements that IVM is ethical [β = -.14, *p* = .039, OR = 0.86, (95% CI, 0.75 to 0.99)], a viable alternative to farmed meat [β = -.15, *p* = .029, OR = 0.85, (95% CI, 0.74 to 0.98)], and that IVM would reduce the impact of global warming associated with farming [β = -.154, *p* = .029, OR = 0.86, (95% CI, 0.75 to 0.98)]. Being politically conservative was more likely to predict agreeing with statements that IVM is unnatural [β = -.14, *p* = .039, OR = 0.86, (95% CI, 0.75 to 0.99)], disrespectful to nature [β = .167, *p* = .024, OR = 1.18, (95% CI, 1.02 to 1.37)], and will reduce the number of happy animals on earth [β = .14, *p* = .041, OR = 1.16, (95% CI, 1.01 to 1.33)].

The higher level of support for IVM shown by political left/liberal participants aligns with general political ideology in which liberal voters tend to be more progressive and focus more on harm and fairness relative to other moral faucets, compared to their conservative counterparts [35, 36]. Therefore, liberal voters might be more willing to engage with IVM as they are more concerned with the issues that is has the potential to address: animal welfare, environmental impact of agriculture, food supply etc.

#### Eating habits

Eating habits were also predictive of a number of responses. Diet categorisation predicted a number of responses. Pescatarians were most likely to perceive IVM to be healthy [β = 1.77, *p* = .026, OR = 5.89, (95% CI, 5.89 to 1.24)] and tasty [β = 1.76, *p* = .034, OR = 5.82, (95% CI, 1.14 to 29.79)] compared to farmed meat. Vegans perceived IVM as more natural than farmed meat [β = 2.01, *p* = .001, OR = 7.46, (95% CI, 2.30 to 24.14)], and both vegetarians [β = -1.94, *p* < .001, OR = 6.95, (95% CI, 2.67 to 18.09)] and vegans [β = -1.94, *p* = .001, OR = 6.99, (95% CI, 2.23 to 21.97)] perceived IVM to be more appealing compared to farmed meat. Willingness to try and eat IVM regularly was found to be lowest for both vegetarians [β = -1.52, *p* = .002, OR = 0.22, (95% CI, 0.08 to 0.56)] and vegans [β = -2.96, *p* < .001, OR = 0.05, (95% CI, 0.02 to 0.17)]. Willingness to eat IVM instead of meat substitutes was also predicted by eating habits, with vegetarians [β = -1.90, *p* = .001, OR = 0.15, (95% CI, 0.05 to 0.47)] and vegans [β = -3.09, *p* < .001, OR = 0.98, (95% CI, 0.01 to 0.18)] being least willing to eat IVM as a replacement. Pescatarians β = 1.99, *p* = .023, OR = 7.29, (95% CI, 1.32 to 40.36)], vegetarians [β = 1.21, *p* = .3.34, OR = 0.59, (95% CI, 1.06 to 10.51)], and vegans [β = 1.80, *p* = .010, OR = 6.10, (95% CI, 1.54 to 24.19)] were also most willing to pay more for IVM relative to farmed meat. Finally, vegans were least likely to agree that IVM is ethical β = -1.5, *p* = .007, OR = 0.21, (95% CI, 0.07 to 0.65), while vegetarians were least likely to agree that IVM could increase the potential for cannibalism, β = 1.3, *p* = .005, OR = 3.92, (95% CI, 1.50 to 10.26).

Eating habits in regards to the percentage of meat consumed was also predictive of a number of responses. People who ate more meat were less likely to perceive IVM as ethical compared to farmed meat [β = -.011, *p* = .007, OR = 0.99, (95% CI, 0.98 to 1.00)], and were also more likely to select unsure in response to whether IVM is more environmentally friendly than farmed meat [β = -.013, *p* = .002, OR = 0.99, (95% CI, 0.98 to 1.00)]. They were also less likely to agree that IVM is ethical [β = -.012, *p* = .002, OR = 0.99, (95% CI, 0.98 to 1.00)]. People who ate more meat were also less likely to think that IVM would reduce the impact of global warming associated with farming, though those who ate the highest percentage of meat were more likely to select unsure β = -.009, *p* = .015, OR = 0.99, (95% CI, 0.98 to 1.00). Finally, in regards to the statement ‘IVM is disrespectful to nature’ a response was identified where strongly agreeing or disagreeing with the statement was predicted by eating the least meat, while those who ate the most meat were more likely to respond in the middle β = .101, *p* = .013, OR = 1.01, (95% CI, 1.00 to 1.02).

These results demonstrate an apparent paradox: those who are already meat restrictive appear less willing to engage with IVM; however, along with pescatarians, these groups generally reported more positive views of IVM compared to farmed meat. This may be reflective of negative perceptions of farmed meat, suggesting that people who already abstain from eating meat may be unwilling to engage with the product, despite awareness of the potential benefits. In contrast, meat eaters, appear more engaged with the product, regardless of their ethical/health perspectives. Moreover, participants who had a higher reported percentage of meat in their diet tended to express less positive views of IVM than those with a lower percentage, however it did not influence willingness to actually engage with the product. This suggests that people who eat more meat may be reluctant to engage with an alternative meat source, such as that produced by IVM, which could be related to an already more positive view of farmed meat.

#### Other demographics

Income was predictive of perceptions of IVM, where participants with higher incomes saw IVM as less ethical β = -.11, *p* = .018, OR = 0.89, (95% CI, 0.81 to 0.98) and were less willing to try IVM β = -.105, *p* = .028, OR = 0.90, (95% CI, 0.82 to 0.99) than those with lower incomes. Neither age nor education level was found to be predictive of any responses.

Overall, these findings demonstrate the complex relationship between people’s own perceptions and behaviours and how this relates to their attitudes towards and willingness to engage with IVM. Attitudes towards IVM were mixed. While a reasonably large proportion of the sample reported willingness to try IVM in the future, there appears to be hesitation around the idea of incorporating into one’s diet regularly. Resistance appears to come primarily from practical restrictions, such as taste and price—factors that are largely non-psychological. These issues will need to be primarily addressed by the market and the product itself. However, naturalness and appeal are also commonly cited concerns. In line with this, research has shown that the largest drops in perceptions of naturalness were brought about by the insertion of a gene from another species relative to other processes such as freezing or adding of synthetic chemicals—a process that is conceptually similar to the production of IVM [37]. Past research hasexamined GM food opposition and the concept of moral disgust in an American sample [24]. The authors found that of the 64% of participants who opposed GM food, the majority could be described as moral absolutists—that is, their opposition to GM was found to be evidence insensitive. Moreover, those who opposed GM food tended to feel heightened disgust towards them. The authors also found that, contrary to previous research [38], participants’ moral absolutism could not be swayed by strong arguments—thus suggesting minimal opportunity for the acceptance of GM foods.

These perceptions could be easily applicable to the concerns of naturalness and appeal surrounding IVM—it could be that those who express concerns around these concepts are evidence insensitive and opposed on similar grounds to those who oppose GM food. Indeed, there is preliminary evidence that the kinds of concerns raised by moral absolutists to GM foods are being raised here also. Future research should examine the potential perceptual links between these and similar high-tech (and thus unnaturally perceived) food products. By determining if moral absolutism is involved in opposition generally we can better understand the why of this process, which is the prequel step to how we might address these concerns to foster societal acceptance.

## Supporting information

S1 Dataset (XLSX)

S1 Questions Full list of questions presented in survey. (DOCX)

S2 Questions Questions in which ‘level of income’ was excluded from the analysis. (DOCX)
