# Supplementary material for: Attitudes to in vitro meat: A survey of potential consumers in the United States
Source: PLoS One. 2017 Feb 16;12(2):e0171904. doi: 10.1371/journal.pone.0171904 (PMC5312878; doi:10.1371/journal.pone.0171904)
Supplement: S1 Questions — (DOCX) [file pone.0171904.s002.docx]

**Full list of questions.**

People's Perceptions of In Vitro Meat

Q62 Attitudes towards Meat Consumption

**The purpose of the study**

This study is examining people's perceptions towards a potential new meat production method and meat consumption in general.

**Risks and benefits of participation**

By participating in this research you are contributing to academic research trying to better understand how the public perceives new potential methods of meat production. There are no risks of participation beyond that of everyday living.

**Participation and withdrawal**

Participation in this survey is entirely voluntary and anonymous and you are able to withdraw at any time.

**What is involved?**

Participation involves responding to online questions. Participation generally takes around 5 minutes. As this survey is looking at your opinions there are no right or wrong answers, so please answer these questions as honestly as possible and if you are unsure please make your best guess. If there are any questions that you would prefer not to answer please leave them blank.

**Confidentiality and security of data**

All data collected in this study will be confidential. Specifically, participants will not be asked to provide their name or any other data that could identify them. All surveys will be numbered and these numbers will not be able to be linked to any individual. The data will be seen only by the chief investigator and the research team and will not be accessible to any individual but the chief investigator and her research team. The data from this study will only be used for research purposes.This study has ethical clearance from the University of Queensland - the ethical clearance number is 16-PSYC-PHD-20-AH.

Q66 I have read and understand the conditions of the study. I understand that participation is completely voluntary and anonymous, and that I am free to withdraw at any time without penalty.

- Yes (2)
- No (1)

If No Is Selected, Then Skip To End of Survey

Q64 I am over 18.

- Yes (2)
- No (1)

If No Is Selected, Then Skip To End of Survey

Q59 Please state your age (in years).

Q2 What is your gender?

- Male (1)
- Female (2)
- Other (please specify) (3) ____________________

Q58 In political matters, people sometimes talk about the "left" and the "right". Where would you place yourself on this scale, generally speaking?

|  | 1 (1) | 2 (2) | 3 (3) | 4 (4) | 5 (5) | 6 (6) | 7 (7) | 8 (8) | 9 (9) |
| --- | --- | --- | --- | --- | --- | --- | --- | --- | --- |
| Left:Right (1) |  |  |  |  |  |  |  |  |  |

Q60 In political matters, values are generally considered either "liberal" or "conservative". Which set of ideas most closely suits your own opinions?

|  | 1 (1) | 2 (2) | 3 (3) | 4 (4) | 5 (5) | 6 (6) | 7 (7) | 8 (8) | 9 (9) |
| --- | --- | --- | --- | --- | --- | --- | --- | --- | --- |
| Liberal:Conservative (1) |  |  |  |  |  |  |  |  |  |

Q4 What is your income per annum?

- Less than $20,000 (1)
- $21,000 - $39,000 (2)
- $40,000 - $59,000 (3)
- $60,000 - $79,000 (4)
- $80,000 - $99,000 (5)
- More than $100,000 (6)

Q5 What is your highest level of completed education?

- No education (1)
- Primary school (2)
- Some high school (3)
- Completed high school (4)
- Technical qualification or trade certificate (5)
- College/Undergraduate degree (6)
- Postgraduate degree (7)

Q6 What are your eating habits?

- Meat-eating (1)
- Eat white meat only (2)
- Pescatarian (3)
- Vegetarian (4)
- Vegan (5)
- Other (please specify) (6) ____________________

Q54 What percentage of your diet is made up of meat?

______ Please drag the bar to the appropriate position (1)

Q7 Have you heard the term in vitro meat before?

- Yes (1)
- No (2)
- Unsure (3)

Q8 Do you know what in vitro meat is?

- Definitely yes (1)
- Probably yes (2)
- I am unsure (3)
- Probably not (4)
- Definitely not (5)

Q31 In vitro meat is an animal flesh product that has never been part of a living animal, but is instead grown in a laboratory using muscle stem cells. These stem cells are extracted without suffering to the animal. In vitro meat is also referred to as cultured meat, schmeat or synthetic meat.   In August 2013, scientists unveiled (and tasted) the world's first in vitro grown hamburger patty. Currently it is not commercially available, though research is being conducted to introduce it as a potential new meat production technique for the future.    ​ The unveiling of the world's first in vitro hamburger in London, August 2013

Q10 The next questions are going to ask about your perceptions of in vitro meat compared to meat that is produced by farming. We will refer to this as farmed meat.

Q11 How healthy do you think in vitro meat is compared to farmed meat?

- Much more healthy (1)
- Somewhat more healthy (2)
- Neither more healthy nor less healthy (3)
- Somewhat less healthy (4)
- Much less healthy (5)

Q12 How natural do you think in vitro meat is compared to farmed meat?

- Much more natural (1)
- Somewhat more natural (2)
- Neither more natural nor less natural (3)
- Somewhat less natural (4)
- Much less natural (5)

Q13 How environmentally friendly do you think in vitro meat is compared to farmed meat?

- Much more environmentally friendly (1)
- Somewhat more environmentally friendly (2)
- Neither more environmentally friendly nor less environmentally friendly (3)
- Somewhat less environmentally friendly (4)
- Much less environmentally friendly (5)

Q14 How ethical do you think in vitro meat is compared to farmed meat?

- Much more ethical (1)
- Somewhat more ethical (2)
- Neither more ethical nor less ethical (3)
- Somewhat less ethical (4)
- Much less ethical (5)

Q17 How appealing do you think in vitro meat is compared to farmed meat?

- Much more appealing (1)
- Somewhat more appealing (2)
- Neither more appealing nor less appealing (3)
- Somewhat less appealing (4)
- Much less appealing (5)

Q18 How tasty do you think in vitro meat would be compared to farmed meat?

- Much more tasty (1)
- Somewhat more tasty (2)
- Neither more tasty nor less tasty (3)
- Somewhat less tasty (4)
- Much less tasty (5)

Q50 How much of a risk do you think there would be for zoonosis (infectious disease transfer from animals to humans) for in vitro meat compared to farmed meat?

- Much more risk (1)
- Somewhat more risk (2)
- Neither more risk nor less risk (3)
- Somewhat less risk (4)
- Much less risk (5)

Q19 For the following questions, please imagine that in vitro meat is commercially available in supermarkets and butchers.

Q23 On a global level, to what extent do you think meeting demand for meat using in vitro methods would be cheaper or more expensive than using farmed meat?

- Much less expensive than farmed meat (1)
- Somewhat less expensive than farmed meat (2)
- Neither more nor less expensive than farmed meat (3)
- Somewhat more expensive than farmed meat (4)
- Much more expensive than farmed meat (5)

Q24 Would you be willing to try in vitro meat?

- Yes, definitely (1)
- Yes, maybe (2)
- Unsure (3)
- No, probably not (4)
- No, definitely not (5)

Answer If Would you be willing to try in vitro meat?  No, definitely not Is Not Selected

Q46 Would you be willing to eat in vitro meat regularly?

- Yes, definitely (1)
- Yes, maybe (2)
- Unsure (3)
- No, probably not (4)
- No, definitely not (5)

Answer If Would you be willing to try in vitro meat?  No, definitely not Is Not Selected

Q47 Would you be willing to eat in vitro meat as a replacement for farmed meat?

- Yes, definitely (1)
- Yes, probably (2)
- Unsure (3)
- No, probably not (4)
- No, definitely not (5)
- Not applicable (I do not currently eat farmed meat) (6)

Answer If Would you be willing to try in vitro meat?  No, definitely not Is Not Selected

Q53 How willing would you be to eat in vitro meat compared to meat substitutes (i.e. made from soy)?

- Much more (1)
- Somewhat more (2)
- Neither more nor less (3)
- Somewhat less (4)
- Much less (5)

Answer If Would you be willing to try in vitro meat?  No, definitely not Is Not Selected

Q22 If you were to buy in vitro meat, how much would you be willing to pay for it compared to farmed meat?

- Much more (1)
- Somewhat more (2)
- Neither more nor less (3)
- Somewhat less (4)
- Much less (5)

Q69 Why might you be unwilling to try in vitro meat? Select all that apply.

- Ethical concerns (1)
- Religious reasons (2)
- Taste/appeal of the product (3)
- Other (please specify) (4) ____________________

Q25 What types of meat do you currently eat? (Please tick all that apply)

- Fish and seafood (1)
- Poultry (2)
- Bacon, ham and/or pork (3)
- Beef (4)
- Horse (5)
- Dog and/or cat (6)
- None (7)

Q43 What types of meat would you be willing to eat if they were produced using in vitro methods? (Please tick all that apply)

- Fish and other seafood (1)
- Poultry (2)
- Bacon, ham and/or pork (3)
- Beef (4)
- Horse (5)
- Dog and/or cat (6)
- None (7)

Q60 Please rate how much you agree with the following statements.

|  | Strongly agree (1) | Somewhat agree (2) | Neither agree nor disagree (3) | Somewhat disagree (4) | Strongly disagree (5) |
| --- | --- | --- | --- | --- | --- |
| In vitro meat is unnatural (1) |  |  |  |  |  |
| In vitro meat is disrespectful to nature (2) |  |  |  |  |  |
| In vitro meat will reduce the number of happy animals on earth (3) |  |  |  |  |  |
| In vitro meat will encourage the possibility that humans could be eaten i.e. cannibalism could occur (11) |  |  |  |  |  |
| In vitro meat is ethical (12) |  |  |  |  |  |
| In vitro meat will improve animal welfare conditions (13) |  |  |  |  |  |
| In vitro meat will be able to solve world famine problems (14) |  |  |  |  |  |
| In the future, in vitro meat will be a viable alternative to farmed meat (15) |  |  |  |  |  |
| In vitro meat will reduce the impact of global warming associated with farming (16) |  |  |  |  |  |
| The production of in vitro meat will have a negative impact on traditional farmers (17) |  |  |  |  |  |

Q40 Do you have any final comments or thoughts that you would like to express about in vitro meat?

**Study Background**

The aim of this survey was to examine the general public's understanding and perceptions of in vitro meat. By understanding how the public feels, we aim to identify any barriers that may restrict the ability for this product to be integrated into society if it becomes commercially available. If you would like to learn more about in vitro meat, please copy and paste this link into a new page your browser: http://culturedbeef.net/what-is-it/

This study has been cleared in accordance with the ethical review processes of the University of Queensland, and within the guidelines of the National Health and Medical Research Council’s guidelines. If you would like to speak to an officer of the University who is not involved in these studies, please contact the School of Psychology Ethics Review Officer directly on 3365 6394 (message on 3365 6230), or contact the University of Queensland Ethics Officer on 3365 3924.
