# Supplementary material for: Attitudes to in vitro meat: A survey of potential consumers in the United States
Source: PLoS One. 2017 Feb 16;12(2):e0171904. doi: 10.1371/journal.pone.0171904 (PMC5312878; doi:10.1371/journal.pone.0171904)
Supplement: S2 Questions — (DOCX) [file pone.0171904.s003.docx]

**Questions for which ‘level of income’ was excluded in the analysis**

Q14 How ethical do you think in vitro meat is compared to farmed meat?

- Much more ethical (1)
- Somewhat more ethical (2)
- Neither more ethical nor less ethical (3)
- Somewhat less ethical (4)
- Much less ethical (5)

Q53 How willing would you be to eat in vitro meat compared to meat substitutes (i.e. made from soy)?

- Much more (1)
- Somewhat more (2)
- Neither more nor less (3)
- Somewhat less (4)
- Much less (5)

Strongly agree (1

| Question | Strongly agree | Somewhat agree | Neither agree nor disagree | Somewhat disagree | Strongly disagree |
| --- | --- | --- | --- | --- | --- |
| In vitro meat is ethical (12) |  |  |  |  |  |
| In vitro meat will improve animal welfare conditions (13) |  |  |  |  |  |
| In vitro meat will be able to solve world famine problems (14) |  |  |  |  |  |
| In the future, in vitro meat will be a viable alternative to farmed meat (15) |  |  |  |  |  |
| In vitro meat will reduce the impact of global warming associated with farming (16) |  |  |  |  |  |
